# Molecular detection of bornavirus in parrots imported to China in 2022

**DOI:** 10.1186/s12917-023-03825-8

**Published:** 2023-12-06

**Authors:** Li-Na Zhang, Yu-Han Huang, Hao Liu, Li-Xia Li, Xue Bai, Guang-Da Yang

**Affiliations:** 1 Eco-Engineering Department, Guangdong, Eco-Engineering Polytechnic, Guangzhou, Guangdong Province China; 2 School of Life Sciences and Engineering, Foshan University, Foshan, Guangdong Province China; 3 Institute of Special Economic Animal and Plant Sciences, Chinese Academy of Agricultural Sciences, Changchun, Jilin Province China; 4 Guangdong Wildlife Rescue Monitoring Center, Guangdong Academy of Forestry, Guangzhou, Guangdong Province China

**Keywords:** Avian bornavirus, Parrot, Parrot bornavirus, Sequence analysis

## Abstract

**Background:**

Avian bornavirus (ABV) is a neurotropic virus, it has been established as the primary causative agent of proventricular dilatation disease (PDD). However, substantial international trade and transnational trafficking of wild birds occur, potentially enabling these birds to harbor and transmit pathogens to domestic poultry, adversely affecting their well-being. Real-time RT-PCR was employed to detect the presence of PaBV-4 in parrots imported to China in 2022.

**Results:**

In 2022, a total of 47 cloacal swabs from 9 distinct species of parrots were collected at the Wildlife Rescue Monitoring Center in Guangdong, China. The purpose of this collection was to detect the presence of PaBV-4. Using real-time PCR techniques, it was determined that the positive rate of PaBV-4 was 2.12% (1 out of 47) in parrots. The PaBV-4 virus was detected in a *Amazona aestiva* that had been adopted for one month. Conversely, all other species tested negative for the virus. Subsequently, the whole genome of the PaBV-4 GD2207 strains was sequenced, and the homology and genetic evolution between these strains and previously published PaBV-4 strains on GenBank were analyzed using DNAStar and MEGA7.0 software. The findings revealed that the full-length genome of PaBV-4 consisted of 8915 nucleotides and encoded six proteins. Additionally, it exhibited the highest nucleotide similarity (99.9%) to the GZ2019 strain, which causes death and severe clinical symptoms in *Aratinga solstitialis*. Furthermore, when compared to other strains of PaBV-4, the GD2207 strain demonstrated the highest amino acid homology with GZ2019. The phylogenetic analysis demonstrated that the GD2207 strain clustered with various strains found in Japanese, American, and German parrots, indicating a close genetic relationship with PaBV-4, but it revealed a distant relationship with PaBV-5 Cockg5 from America. Notably, the GD2207 was closely associated with the GZ2019 strain from *Aratinga solstitialis* in China.

**Conclusion:**

This study presents the preliminary identification of PaBV-4 in *Amazona aestiva* parrots, emphasizing its importance as the predominant viral genotype linked to parrot infections resulting from trade into China. Through genetic evolution analysis, it was determined that the GD2207 strain of PaBV-4 exhibits the closest genetic relationship with GZ 2019 (*Aratinga solstitialis*, China), M14 (Ara macao, USA), AG5 (Psittacus erithacus, USA) and 6758 (*Ara ararauna*, Germany) suggesting a shared ancestry.

## Introduction

Avian Bornaviruses (ABV) are characterized as non-segmented, negative-strand RNA viruses, with virions displaying a spherical morphology and measuring approximately 90 nm in diameter [1, 2]. The genome can encode six distinct proteins, namely nucleoprotein (N), phosphoprotein (P), X protein overlapping with phosphoprotein (X), matrix protein (M), glycoprotein (G), and RNA-dependent RNA polymerase (L). ABV was initially recognized as the etiological agent responsible for fatal adenopathy PDD in 2008 [3, 4]. Additionally, this virus has been detected in waterfowl and songbirds [5–9]. To date, a minimum of 20 distinct ABVs have been identified and classified into five separate species according to a recent reclassification: Passeriform 1 orthobornavirus, Passeriform 2 orthobornavirus, Psittaciform 1 orthobornavirus (including the parrot-bornaviruses PaBV-1, -2, -3, -4 and -7), Psittaciform 2 orthobornavirus (comprising PaBV-5) and Waterbird 1 orthobornavirus. Munia bornavirus 1 (MuBV-1) and the parrot bornaviruses PaBV-6 and-8 remained unclassified [10]. Furthermore, eight distinct ABV genotypes have been identified across more than 80 avian species and 50 parrot species [11–15]. ABV infection primarily manifests in sick birds through symptoms such as abdominal enlargement, decreased body mass, indigestion, ataxia, and other debilitating effects, often leading to a high fatality rate. This poses a significant risk to the health of captive birds, including those belonging to various endangered species [16, 17]. Presently, no efficacious treatment approaches are available for managing ABV infection, with only symptomatic treatment and management being employed [13, 18].

There is a scarcity of reports on the transmission of PaBV-4 through transnational parrot trading to China. This study aims to examine PaBV-4 infection in various parrot species in transnational wildlife imports, utilizing real-time PCR. The outcomes of this investigation aim to provide a point of reference for animal conservation efforts and mitigate the spread of PaBV among birds and poultry.

## Materials and methods

### Sample collection

The Wildlife Rescue Monitoring Center is responsible for receiving, rescuing, domesticating, and breeding animals acquired through donations or fines, including those injured. The ultimate goal is to release them back into their natural habitats. Notably, Pangolins and endangered avian species, particularly parrots, are among the animals rescued from southeast Asia for import or smuggling activities. The aviary accommodates diverse parrot species, each residing in separate cages to ensure no contact between them. Cloacal swabs are obtained from each parrot using sterile cotton swabs and subsequently preserved in tubes containing phosphate-buffered saline (PBS). A total of 47 anal swab samples were collected from 9 species of parrots as follows: Monk Parakeet (7), Sun Parakeet (6), Cockatiel (7), Senegal Parrot (6), Grey Parrot (8), Yellow-crested Cockatoo (2), Eclectus Parrot (6), Amazona aestiva (1), and Blue-winged Macaw (4).

### Real-time PCR detection

The cloacal swabs samples underwent real-time PCR analysis using the previously described specific primers for PaBV-4 [3]. Before this, the laboratory confirmed the absence of PaBV-4 positive samples, supporting the conclusion that the samples were contamination-free. The primers were designed to amplify the whole genome sequence of PaBV-4 (Table 1). The viral RNA extraction was performed from cloacal swabs using the RNeasy Mini Kit (Qiagen, Hilden, Germany) according to the manufacturer's instructions. The PCR products were examined by agarose gel electrophoresis, purified using a QIAquick Gel Extraction Kit (Qiagen, USA), and then sequenced. The complete sequence was submitted to Genbank.

**Table 1:** Oligonucleotide sequences of primers used in study of a PaBV4 isolated from Amazona aestiva, China, in 2022

Primer name	Length of amplification	Sequence (5’-3’)
PaBV4 F1	1107 bp	TGTTGCGGTAACAACCAACCA
PaBV4 R1		TCATCACCACGAGTTATTTCT
PaBV4 F2	1465 bp	GTCAACAATACAACCAGGATC
PaBV4 R2		GCTGTAACTACCAAAGAACCC
PaBV4 F3	1505 bp	GTGTCATGCTATAAGTACCAC
PaBV4 R3		AACTCTAACTATTGTCTCAGC
PaBV4 F4	1548 bp	GATCTTCCGTCGTGTGTCTCT
PaBV4 R4		CCTGTCCTGAAGAAAAACCCT
PaBV4 F5	1636 bp	TCTTGATTACAGTTCTTGGTG
PaBV4 R5		AATCAGCGTATCTCTAACAAG
PaBV4 F6	1098 bp	CAAACACTGCCATAAAGGTCC
PaBV4 R6		TTTCATTACAATGGGCTGTTG
PaBV4 F7	1091 bp	CTTGCACAGGCGGAGGTATCA
PaBV4 R7		GCGCTACAACAAAGCCACAAA

### Sequence alignment and phylogenetic analysis

The analysis involved the examination of genetic variation using complete PaBV 1–7 sequences available in GenBank. Nucleotide sequence alignments and homology comparisons were conducted using the Clustal W method in the MegAlign software. (DNASTAR, Madison, USA).

The phylogenetic analysis focused on the complete coding sequence of the GD2207 strain, comparing it with sequences from 26 other reference genomes from the PaBV1-5 subfamily (Table 2). The Maximum Likelihood (ML) method in MEGA v7.0 was employed to generate various phylogenetic trees based on the aligned nucleotide sequence [19].

**Table 2:** The PaBV sequences obtained from Genbank database for analysis of complete coding sequence

Strain	Virus type	GenBank	Year	Host	Country
1034–1322	PaBV-4	FJ169441.1	2008	Canindae Macaw	USA
2011 Japan	PaBV-5	LC120625.1	2012	Electus roratus	Japan
2014-A	PaBV-5	NC039190.1	2014	Ara ararauna (blue-and-yellow macaw)	Hungary
6609	PaBV-2	FJ620690.1	2008	Amazona ventralis	Australian
6758	PaBV-4	JX065209.1	2008	Ara ararauna	Germany
16021	PaBV-5	MH559279.1	2016	Ara ararauna (blue-and-yellow macaw)	Thailand
16234	PaBV-1	JX065207.1	2011	Nestor notabilis	Germany
16667a	PaBV-7	JX065210.2	2010	Cacatua moluccensis	Germany
AG5	PaBV-4	GU249596.2	2008	Psittacus erithacus	America
AR18A	PaBV-4	LC486412.1	2018	Ara ararauna	Japan
bil	PaBV-2	EU781967.1	2006	Aratinga solstitialis	America
Cockg5	PaBV-5	KT378600.1	2015	cockatoo	America
GD2207	PaBV-4	OQ428243	2022	Amazona aestiva	China
GZ 2019	PaBV-4	MT258650.1	2019	Aratinga solstitialis	China
M14	PaBV-4	JN035149.1	2009	Ara macao	USA
M15	PaBV-4	JN014950.1	2009	Ara ararauna (Blue & Yellow Macaw)	USA
M25	PaBV-1	NC039189.1	2008	Diopsittaca nobilis	America
NM01	PaBV-4	JN035148.1	2009	Aratinga jandaya	USA
NM06	PaBV-4	JN014948.1	2009	Cacatua goffini (Goffin's Cockatoo)	USA
NM20	PaBV-4	JN014949.1	2009	Ara ararauna (Blue & Yellow Macaw)	USA
No.6	PaBV-4	LC486415.1	2018	Poicephalus robustus	Japan
No.18	PaBV-4	LC486416.1	2018	Psittacus erithacus	Japan
No.27	PaBV-4	LC486417.1	2018	Aratinga jandaya	Japan
NTUCL7	PaBV-4	OM939725	2018	—	Taiwar

## Results

Real-time RT-PCR analysis confirmed the presence of PaBV-4 infection in only one Amazona aestiva. It should be noted that each species of parrot was housed in separate, interconnected cages. Although the Amazona aestiva parrot had been housed in the aviary for one month, the other parrot species did not have direct contact with it, and therefore, PaBV-4 was not detected in those parrots. The complete genome of the GD2207 strain (GenBank accession no. OQ428243) spans 8915 bp and encodes various proteins, including N, X, P, M, G, and L, which are consistent with other PaBV-4 strains derived from parrots [20].

### Sequential analysis

The multiple sequence alignments of the complete genome of GD2207 and 23 other reference genomes obtained from the PaBV 1–7 subfamily revealed nucleotide-level similarities ranging from 64.7% to 99.9%. Specifically, the complete genome of the GD2207 strain exhibited a 99.9% identity to the GZ 2019 strain, which was isolated post-mortem from *Aratinga solstitialis* (China, 2019). Additionally, it showed 99.7% identity to M15, NO 18, and NO 6, which were derived from the brain of the *Ara ararauna* (USA, 2009), fecal samples of *Psittacus erithacus*, and *Poicephalus robustus* (Japan, 2018), respectively. However, it exhibited the lowest identity to the PaBV-5 strain, with 64.7% in the 2011/Japan strain (Japan, 2012) and the Cockg5 strain (USA, 2015).

Furthermore, upon comparing the individual proteins of GD2207 with other reference genomes, it was observed that there was a significant amino acid similarity with the N ranging from 75.0% to 100%, X ranging from 55.2% to 100%, P ranging from 69.3% to 100%, M ranging from 86.0% to 100%, G ranging from 68.2% to 99.8%, and L ranging from 66.5% to 100%. Notably, the highest amino acid identity was observed with the GZ 2019 strain (99.8%-100%), followed by M15 (99.5%-100%), NO 18 (98.6%-100%), and NO 6 (98.6%-100%) strains of the PaBV-4 virus individual proteins (Table 3).

**Table 3:** Nucleotide and amino acid sequences and identity analysis of GD2207 and the other PaBV strains

Strains	GD2207 (%)												
	Complete genome	Nucleoprotein (N)		X protein (X)		Phosphoprotein (P)		Matrix protein (M)		Glycoprotein (G)		Viral polymerase (L)	
	nt	aa	nt	aa	nt	aa	nt	aa	nt	aa	nt	aa	nt
1034–1322	99.6	100	99.4	100	100	99.5	99.8	100	99.5	99.8	99.7	99.9	99.7
2011 Japan	64.7	75.5	68.2	55.2	74.7	69.3	71.1	86.7	75.5	68.2	66.8	67.4	62.7
2014-A	65.0	75.0	68.4	55.2	73.9	69.3	70.6	86.7	75.3	68.2	67.4	67.6	63.2
6609	80.6	95.2	81.9	79.3	88.5	95.0	85.1	97.2	86.7	89.8	81.3	90.0	79.3
6758	99.6	100	99.3	98.9	99.6	99.5	99.2	100	99.5	99.8	99.7	99.9	99.7
16,021	65.2	75.3	68.1	58.6	76.6	69.8	72.4	86.0	75.83	69.0	66.6	67.2	63.3
16,234	81.2	94.4	82.0	82.8	90.4	94.6	84.3	97.9	86.5	89.0	81.5	91.3	80.6
16667a	77.8	93.0	80.0	73.6	85.4	93.6	81.4	94.4	85.1	86.4	78.0	86.6	76.7
AG5	99.3	99.7	99.0	100	100	99.5	99.3	100	99.5	99.4	99.5	99.8	99.7
AR18A	99.6	100	99.5	98.9	99.6	99.5	99.5	100	99.5	99.6	99.7	99.8	99.7
bil	80.5	95.2	81.7	78.2	88.9	94.6	84.5	97.2	86.9	89.2	80.9	90.2	79.4
Cockg5	64.7	75.3	68.3	55.2	73.9	69.3	70.8	86.0	74.4	68.4	66.4	66.5	62.7
GZ 2019	99.9	100	99.6	100	100	100	100	100	99.8	99.8	99.9	100	100
M14	99.6	100	99.1	100	100	99.5	99.7	100	99.5	99.8	99.7	99.7	99.7
M15	99.7	100	99.2	100	100	99.5	99.7	100	99.5	99.8	99.7	99.9	99.8
M25	81.1	94.9	82.2	85.1	90.4	94.6	84.5	96.5	86.2	89.8	81.2	90.9	80.1
NM01	94.8	99.2	94.7	93.1	96.6	98.5	95.4	97.9	95.3	97.4	94.2	98.2	95.1
NM06	94.7	98.7	94.4	92.0	96.2	98.5	95.2	98.6	95.6	97.2	94.3	98.1	95.1
NM20	99.6	100	99.1	100	99.6	99.0	99.5	100	99.5	99.8	99.6	99.8	99.8
No.6	99.7	100	99.3	98.9	99.6	99.5	99.7	100	99.3	99.6	99.7	99.9	99.9
No.18	99.7	100	99.3	98.9	99.6	99.5	99.7	100	99.3	99.6	99.7	99.9	99.8
No.27	99.6	100	99.3	98.9	99.6	99.5	99.7	100	99.1	99.4	99.5	99.7	99.8
NTUCL7	96.2	99.5	95.5	94.3	96.6	98.5	96.2	99.3	97.0	97.8	96.4	98.8	96.4

### Genetic evolution analysis

**Fig. 1:**
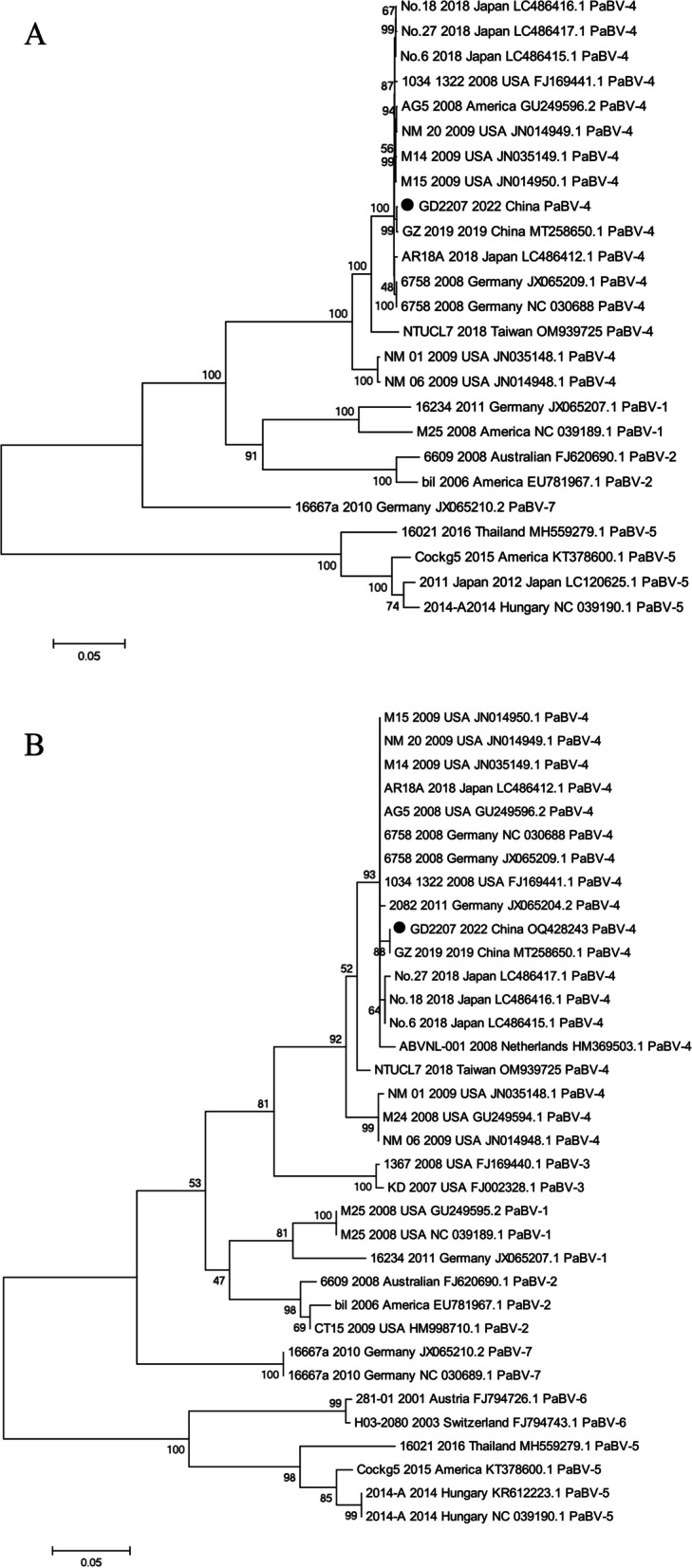
Phylogenetic analyses were conducted on the whole genome sequences (**A**) and M gene sequences (**B**) of PaBV-4, which were detected from the *Amazona aestiva* in 2022. The evolutionary history was determined using the Maximum Likelihood method in MEGA version 7. The bootstrap test, conducted with 1000 replicates, determined the percentage of replicates wherein the associated virus clustered together, and this information is displayed next to each tree branch. Each node's bootstrap support percentage is represented by a specific value, and the strain isolated in this study is denoted by black circles

The whole genome and M gene sequence of PaBV 1–7 obtained in this study, along with PaBV sequences of 24 strains downloaded from GenBank, were analyzed using MEGA 7 software (Fig. 1A and B). The findings showed that the GD2207 revealed a close genetic relationship with GZ 2019 (*Aratinga solstitialis*, China), M14 (*Ara macao*, USA), AG5 (*Psittacus erithacus*, USA), and 6758 strains (*Ara ararauna*, Germany), as they belonged to a small clade (Fig. 1B).

## Discussion

ABV can potentially infect various avian species, including parrots, canaries, and other birds, resulting in a high fatality rate. Previous research has identified ABV infections in various avian species, such as toucans, Canada geese, ostriches, ducks, owls, flocks, finches, sparrows, and other passerine birds, as documented in the literature. Initially discovered in parrots, ABV has demonstrated the ability to infect many parrot species. Several studies have indicated that ABV can be effectively excreted through various secretions, including feces, urine, and even feather pollutants, which are considered potential sources of contamination [8, 14]. PaBV exhibits high pathogenicity towards various species of parrots, posing a significant threat to their populations. The clinical symptoms associated with PaBV infection include central nervous signs like seizures, opisthotonus, ataxia, tremors, or central blindness, and gastrointestinal signs like diarrhea with undigested seeds in the feces, dilated proventriculi. Additionally, affected birds may exhibit intermittent regurgitation, vomiting, polydipsia, and polyuria, along with non-specific symptoms such as general depression and weight loss, which can lead to death [21–23]. There have been limited reports to date of PaBV-4 infection in parrots imported to China. A Amazona aestiva that had been transported to China was housed in a segregated enclosure within the avian facility of the Wildlife Rescue Center, where the presence of PaBV-4 was identified using the real-time PCR technique. Rescued birds, whether reintroduced into their native environment or adopted, pose a potential risk of transmitting the virus to other avian species and domestic poultry. Consequently, the trade of parrots have the potential to amplify the transmission of PaBV further. However, isolating infected birds may serve as a preventive measure against transmitting PaBV.

The whole genome of the GD2207 strain is 8915 nucleotides long and encodes 6 different proteins, exhibiting similarity to PaBV-4 strains GZ2019, M15, NO 18, and NO 6, which were detected in parrots. The homology analysis revealed nucleotide similarities of 94.7% to 99.9% and amino acid similarities of 98.0% to 99.8% between the GD2207 strain and other reference strains of PaBV-4, indicating a high degree of conservation. The highest nucleotide and amino acid homology was observed in the GZ 2019 strain (Table 3). The GZ 2019 strain has been reported to cause death and severe clinical symptoms with *Aratinga solstitialis*. The analysis of nucleic acid sequences revealed a nucleotide similarity of 99.9% between the GD2207 and GZ2019 strains. Notably, variations were observed in the Nucleoprotein, Matrix protein, and Glycoprotein genes, with only the Glycoprotein gene exhibiting gene mutations that impacted the amino acid sequence. The alterations in nucleotide and amino acid sequences did not result in an elevated level of pathogenicity in the GD2207 strain, which was observed in Amazona aestiva birds exhibiting subclinical symptoms. Genetic analysis suggests that the GD2207 strain shares a close genetic relationship with GZ 2019, M14, AG5, and 6758, potentially evolving from a common ancestor. However, additional analyses are necessary to determine if the GD2207 strain can cause mortality or severe clinical symptoms in other avian species.

In summary, this study provides the recorded instance of PaBV-4 infection in Amazona aestiva parrots obtained from imported birds. The GD2207 strain examined in this study exhibits a close genetic relationship with PaBV-4 strains previously identified in China, America, Germany, Japan, and the Netherlands. The global dissemination of the virus may be attributed to trade or illicit trafficking. Furthermore, it is recommended that future research should focus on detecting PaBV-4 infection in wild birds and domestic fowl during commercial transactions or illicit trafficking. Concurrently, quarantining infected birds in individual enclosures should be implemented, as this measure will effectively mitigate the dissemination of the viral agent.

## Data Availability

All data and materials are within this published paper. The datasets generated and/or analysed during the current study are available in the NCBI GenBank database repository OQ428243.

## Abbreviations

ABV: Avian Bornavirus
PDD: Avian glandular dilation disease
PaBV: Parrot Bornavirus
MuBV-1: Munia bornavirus 1
